# Incidental finding of acquired cystic kidney disease associated renal cell carcinoma in an adolescent with kidney failure

**DOI:** 10.1007/s00467-024-06364-y

**Published:** 2024-04-10

**Authors:** Alice Ming-jie Chuah, Eugene Yu-hin Chan, Pak-chiu Tong, Alison Lap-tak Ma

**Affiliations:** 1 Paediatric Nephrology Unit, Woman and Children’s Hospital, Kuala Lumpur, Malaysia; 2 Paediatric Nephrology Centre, Hong Kong Children’s Hospital, Kowloon, Hong Kong SAR; 3 Department of Paediatrics, Chinese University of Hong Kong, Shatin, Hong Kong SAR

**Keywords:** Acquired cystic kidney disease, Renal cell carcinoma, Kidney failure, Dialysis

## Abstract

Acquired cystic kidney disease (ACKD) can occur in patients with chronic kidney disease and kidney failure, and its incidence increases with the duration of dialysis. In adults, ACKD is less common in the pre-dialysis group (~ 7%), but its incidence can be as high as 80% for those who are on dialysis for more than ten years. There is, however, very little information about the prevalence of ACKD in children. We report a case of malignant transformation of ACKD following a kidney transplant, highlighting the importance of surveillance of the native kidneys in paediatric patients who have been in long-term kidney replacement therapy.

## Case summary

A 12-year-old boy of Pakistani ancestry was referred to our unit for management of kidney failure. His parents are first-degree cousins, and he has two cousins who suffered from kidney failure due to an unknown primary kidney disease. Our patient presented with a 1-month history of fatigability, anorexia, vomiting, and weight loss. He was hypertensive at presentation with markedly elevated urea (27.4 mmol/L) and creatinine (827 µmol/L) levels. His ultrasound showed bilateral atrophic kidneys, with an increase in renal parenchymal echogenicity and loss of corticomedullary differentiation. There was no kidney cyst. Whole exome sequencing (WES) did not reveal any pathological variants. He was commenced on automated peritoneal dialysis (APD) soon after admission and enlisted for transplant.

The patient was doing relatively well on PD until the fourth year into the treatment. He was admitted on and off for abdominal pain. Blood results were unremarkable, and peritonitis was ruled out. An urgent abdominal ultrasound (USS) showed cystic changes over bilateral kidneys, with the largest cyst being 2 cm × 1 cm and no mention of complex cysts. He was discharged with supportive care but returned again with acute onset of left loin pain with fever a few months later. Urgent computed tomography (CT) scan of the abdomen demonstrated a ring-enhancing lesion at the upper pole of the left kidney (5.0 cm × 4.4 cm × 4.8 cm), suggesting an infected kidney cyst with abscess formation. He underwent percutaneous CT-guided drainage of the infected cyst together with intravenous piperacillin/tazobactam for a total of 14 days. The culture of abscess aspirate was negative. He responded well to antibiotics with complete resolution of the abscess upon repeated imaging.

**Fig. 1:**
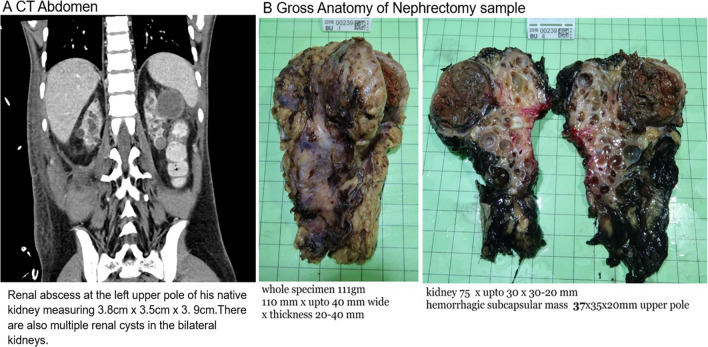
**A**) Urgent contrast-enhanced CT scan of the abdomen showing kidney abscess over the upper pole of the left native kidney in the background of ACKD; and **B**) Goss examination of the nephrectomized left native kidney revealed multiple kidney cysts with a well encapsulated subcapsular haemorrhagic roundish mass, subsequently confirmed to be ACKD-associated renal cell carcinoma

One month following his recovery, he received a deceased donor kidney transplant. He was induced with intravenous methylprednisolone and basiliximab, followed by triple maintenance immunosuppression with prednisolone, tacrolimus, and mycophenolate mofetil. The transplant was uneventful with good initial graft function. On the third day post-transplant, he developed clinical sepsis and severe hypertension. He was admitted to the intensive care unit for further management, including intravenous labetalol infusion for blood pressure control. His total white blood cell count was 3.7 × 10^9^/L (normal range 3.94–9.67 × 10^9^/L), C-reactive protein 17 mg/L (normal range 1–8 mg/L), procalcitonin 1.05 ng/ml (normal value < 0.5 ng/ml), and blood culture was positive for Klebsiella pneumoniae. Urgent contrast CT scan of the abdomen revealed a kidney abscess over the upper pole of the left native kidney (Fig. 1A). Urgent left native nephrectomy was done on the same day (Fig. 1B). The pathology report suggested stage I ACKD-associated renal cell carcinoma (RCC) (T1N0M0). Subsequently, a right native kidney nephrectomy was also performed, and no evidence of malignancy was found on the right kidney. The patient was discharged 3 weeks after the transplant. At his last follow-up, his serum creatinine was static at 110 µmol/L with eGFR of 75 ml/min/1.73 m^2^.

## Discussion

ACKD is an important but often neglected complication in patients with kidney failure. Although the exact pathogenesis remains unclear, uraemia was hypothesized to be the primary mediator. These cysts may regress after a kidney transplant. Patients with ACKD are usually asymptomatic, and the lesions are often detected incidentally. In adult patients, the minimal number of cysts required to diagnose ACKD is three in each kidney, but this may be debatable in children [1, 2]. The common manifestations of ACKD include gross haematuria, loin pain, or infections. However, serious complications such as rupture of the cystic blood vessels leading to catastrophic bleeding and malignant transformation were also reported [3]. The incidence of RCC in ACKD is often underestimated as most of them are asymptomatic or only detected incidentally. The incidence of RCC as a complication of ACKD varies in different cohorts, but patients with ACKD had a 100-fold increased risk to develop RCC compared to the general population [4]. Fortunately, death from metastatic RCC among dialysis patients is uncommon.

As mentioned, in view of the potential sinister complications of ACKD, pre-emptive detection and early intervention is advisable. Meticulous monitoring is warranted in patients presenting any symptoms associated with ACKD or alterations in the cystic patterns. To date, there is no consensus on the surveillance protocol for ACKD in patients with kidney failure. USS could be a valuable screening tool as it is available, non-invasive, and without radiation. However, USS is operator-dependent and might be less sensitive to detect small cysts or cysts with complex architecture. Alternative imaging modalities such as CT scan and magnetic resonance imaging (MRI) might provide better characterisation for complex cysts. The Bosniak classification has been widely used in the classification of cystic changes in ACKD detected by CT scan [1].

In our patient, he developed ACKD after 4 years of dialysis. In contrast to European countries such as the UK, the waiting time to transplant is rather long in some parts of Asia, including Hong Kong. Patients are therefore particularly susceptible to developing ACKD [5]. Importantly, although annual USS confirmed the diagnosis of ACKD, it failed to demonstrate the features of complex cyst, which turned out to be malignant. This highlights the limitation of USS as the first-line screening tool. CT scans are more sensitive, but repeated scanning could be associated with significant radiation exposure. MRI with gadolinium contrast is generally not recommended in patients with kidney failure due to the potential complication of nephrogenic systemic fibrosis. Further studies are required to define the best imaging modality for screening and characterisation of the cysts.

## Conclusions

This case illustrated the imminent need for effective surveillance protocol for early detection of ACKD and its complications in children with kidney failure. A large-scale global study would be of great value to determine the incidence, demographics, and the prognosis of ACKD in the paediatric population.

## Summary

### What is new?

- Although most patients with acquired cystic kidney disease are asymptomatic, serious complications such as renal cell carcinoma may arise. Clinicians should be alert to this phenomenon, and close monitoring is advised for children who are at risk.
